# Supplementary material for: Identification of photoperiod-induced specific miRNAs in the adrenal glands of Sunite sheep (Ovis aries)
Source: Front Vet Sci. 2022 Jul 22;9:888207. doi: 10.3389/fvets.2022.888207 (PMC9354845; doi:10.3389/fvets.2022.888207)
Supplement: Supplementary file 1 [file Data_Sheet_1.ZIP › Supplementary file/miRDeep2 file.zip!/miRDeep2 file/Novel_Structure/SPLP42A3/Novel_519.pdf]

Provisional ID	:	NC_019470.2_23518
Score total	:	1.6
Score for star read(s)	:	-1.3
Score for read counts	:	0
Score for mfe	:	1.3
Score for randfold	:	1.6
Score for cons. seed	:	
Total read count	:	16
Mature read count	:	14
Loop read count	:	2
Star read count	:	0

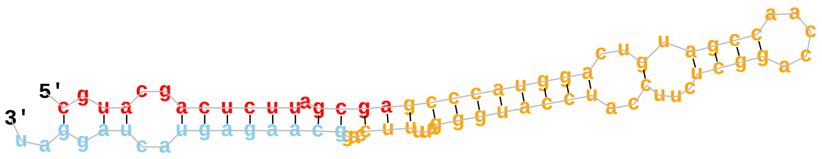

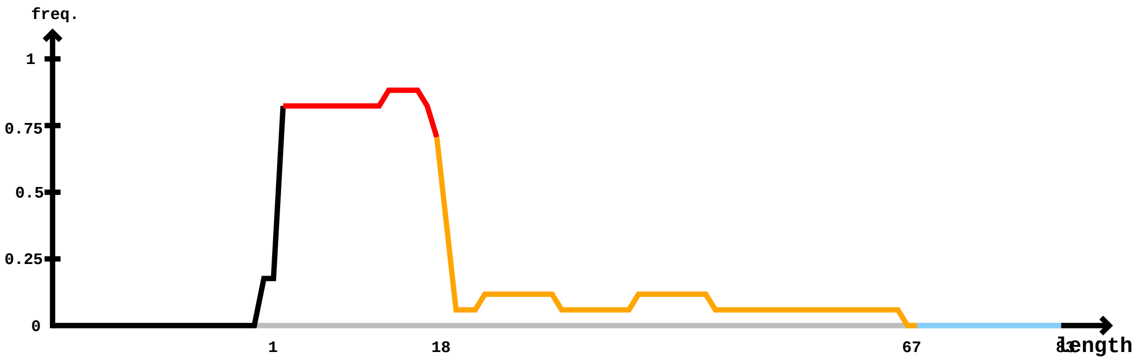


```
                             Mature                                            Star
5'- ugaaagugaagucgcucagucacguacgacucuuagcgagcccauggacuguagccaaccaggcucuuccauccaugggauuuuccaggcaagaguacuaggaugacu   -3'    exp
    ....((((....)))).((((((.((..((((((.(((((((((((((..(.((((.....))))...)..))))))))...)))...))))))))..)).).)))))   reads   mm   sample
    ....................cacguacgacucuuagc.......................................................................   1       0    seq
    ....................cacguacgacucuuagcg......................................................................   2       0    seq
    ......................cguacgacucuuagcga.....................................................................   9       0    seq
    ......................cguCcgacucuuagcga.....................................................................   2       1    seq
    .................................uagcgaUcccauggacug.........................................................   1       1    seq
    ...........................................auggacuguagccUaccaggcucu.........................................   1       1    seq
    ...........................................................caggcucuuccauccaugggauuuucca.....................   1       0    seq
```
